# Clinical performance of a conometric retention system for single implant restorations: 1-year analysis of a prospective multicenter study

**DOI:** 10.1186/s40729-026-00708-z

**Published:** 2026-08-19

**Authors:** Peter Gehrke, Octavio Weinhold, Pelayo Antuna, Anthony Bendkowski, Maria Luisa Broseta, Ernest Cholakis, Lyndon Cooper, Thomas J. McGarry, Martin Wanendeya, Juliane Kreis, Marco Toia

**Affiliations:** 1 Department for Oral, Cranio-Maxillofacial and Facial Plastic Surgery, Goethe University, University Hospital Frankfurt, Frankfurt am Main, Germany; 2 Private Practice for Oral Surgery and Implant Dentistry, Academic Teaching and Research Institution of the Johann Wolfgang Goethe University Frankfurt am Main, Ludwigshafen, Germany; 3 Clinica Dental Antuña de Alaiz, Oviedo, Spain; 4 The Implant Experts, Maidstone, UK; 5 Clinica Dental Broseta, Cheste, Spain; 6 Cholakis Dental Group, Winnipeg, Canada; 7 University of Illinois College of Dentistry Administration, College of Dentistry, Chicago, USA; 8 Implant & Prosthodontic Associates, Oklahoma City, USA; 9 Ten Dental, London, UK; 10 Private Practice, Münster, Germany; 11 Department of Oral and Maxillofacial Surgery and Oral Medicine, Faculty of Odontology, Malmö University, Malmö, Sweden; 12 Studio Toia, Busto Arsizio, Italy

**Keywords:** Conometric retention, Friction-fit abutment, Single implant crown, Implant-supported restoration, Marginal bone level, Peri-implant disease, Prosthetic complications

## Abstract

**Purpose:**

Conventional screw- and cement-retained single implant restorations are associated with biological and technical complications. Friction-retained conometric concepts have been proposed as an alternative; however, clinical evidence for single crowns, particularly from prospective multicenter studies, remains limited. This study evaluated the 1-year clinical performance of a conometric retention system for implant-supported single crowns.

**Methods:**

This prospective multicenter study was conducted at nine clinical centers in Europe and North America. Subjects requiring single-tooth replacement in the maxilla or mandible received one implant restored with a prefabricated 5.9° conometric abutment and a titanium nitride–coated coping. All-ceramic crowns were extraorally bonded and seated without cement or screws. Radiographs were obtained at implant placement, delivery of the permanent restoration, and after 1 year. Primary outcomes were implant and prosthetic survival. Secondary outcomes included marginal bone level (MBL) changes, bleeding on probing, plaque, probing pocket depth, and complication rates. Descriptive statistics and confidence intervals were calculated.

**Results:**

A total of 144 implants were included in the per-protocol analysis. Three implants failed after delivery of the permanent restoration, resulting in a 1-year implant survival rate of 96.7%. Of 131 restorations evaluated at 1 year, 4 showed irreversible loss of conometric retention and were classified as prosthetic failures. In total, 22 prosthetic complication events were recorded, including 18 reversible technical complications (12.5%) and 4 irreversible failures, corresponding to an overall complication rate of 15.3%. Mean MBL change was − 0.11 mm from implant placement to PR and − 0.02 mm from PR to 1 year.

**Conclusions:**

Within the limitations of this 1-year analysis, the investigated conometric retention system demonstrated high implant and prosthetic survival rates and limited marginal bone changes. However, a clinically relevant incidence of technical complications, including irreversible loss of conometric retention, was observed during the first year of function. These findings suggest that the conometric concept may require careful case selection and clinical monitoring. Long-term follow-up is required to further evaluate the clinical stability and prosthetic reliability of this retention concept over time.

*Trial registration*: ClinicalTrials.gov, ID, NCT04063878, August 20, 2019.

## Introduction

Implant-supported single crowns are commonly retained either by screws or by cement. Both approaches are clinically established but associated with specific biological and technical complications. Reported technical events include abutment screw loosening or fracture, loss of retention, ceramic chipping, and marginal discrepancies at the abutment–crown interface [1–7]. Cement-retained restorations, in turn, have been linked to excess cement, peri-implant mucositis, and peri-implantitis even when meticulous removal procedures are followed [8–13]. Overall, no clear superiority has been demonstrated between screw- and cement-retained single crowns with regard to survival or complication-free outcomes [7, 14, 15].

Friction-based double-crown concepts, including telescopic and conometric systems, represent an alternative retention mechanism. In conventional prosthodontics, telescopic crowns supported by natural teeth have achieved favorable long-term outcomes with low complication rates [16, 17]. For implant-supported full-arch prostheses, telescopic and conometric solutions have similarly shown high prosthetic survival and stable function over extended follow-up periods [18–22].

Conometric retention concepts aim to combine selected advantages of cement- and screw-retained restorations by providing a cement-free and screw-free restoration with retrievability and a seamless occlusal surface without interruption of the veneering material. This design may reduce biological complications related to cement residues as well as mechanical complications associated with access holes and veneering fractures while maintaining prosthetic retrievability. However, successful clinical application requires precise seating of the prosthetic components, sufficient restorative space, and meticulous prosthetic execution. In addition, recent clinical data have indicated a higher incidence of prosthesis detachment compared with screw-retained restorations [23].

Conometric engagement is based on a friction fit between a tapered abutment and a corresponding coping. The mechanical stability of this interface depends on factors such as material properties, surface characteristics, and the precision of the cone angle [24]. While this principle has been successfully translated into implant prosthodontics, clinical evidence for conometric retention in single implant restorations remains limited [25], with a notable lack of prospective multicenter data. Beyond a few experimental and single-center reports, robust clinical evidence is still lacking, particularly regarding peri-implant tissue responses, marginal bone level (MBL) changes, and the range of biological and technical complications [26–28].

Therefore, the present study reports the 1-year outcomes of a 5-year prospective multicenter investigation on single implant crowns retained by a dedicated conometric abutment system. The primary aim was to evaluate implant and prosthetic survival and success. Secondary aims were to assess marginal bone level (MBL) changes, peri-implant soft tissue conditions, and the incidence of biological and technical complications.

It was hypothesized that the conometric retention system would demonstrate survival rates, peri-implant tissue conditions, and complication rates comparable to those reported for conventional screw- and cement-retained single implant restorations during the first year of function.

## Materials and methods

### Study design and setting

**Fig. 1:**
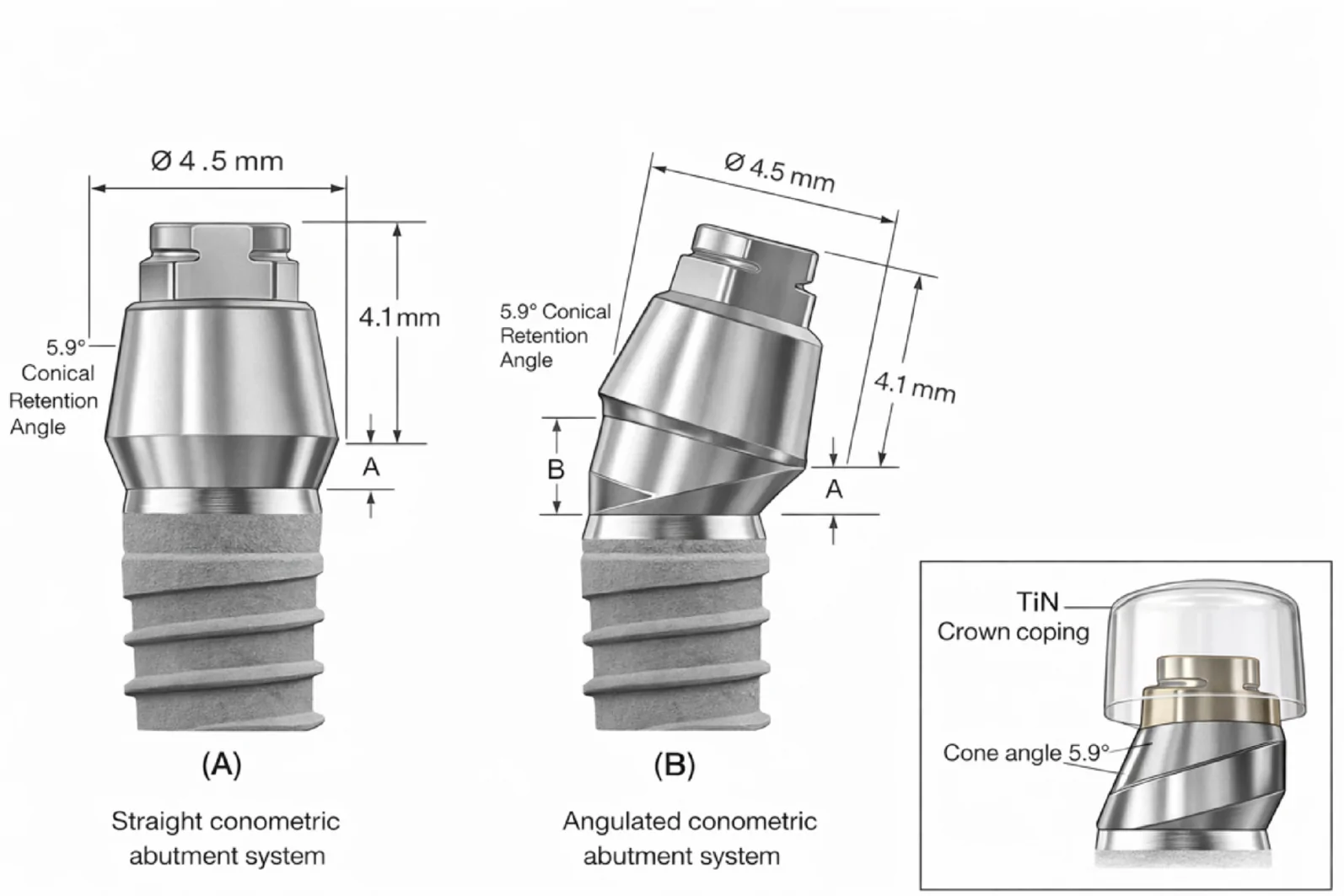
Technical design of the Acuris conometric abutment system, exemplarily illustrated on a XiVE implant, showing straight and angulated abutments and the cone-in-cone retention interface

**Fig. 2:**
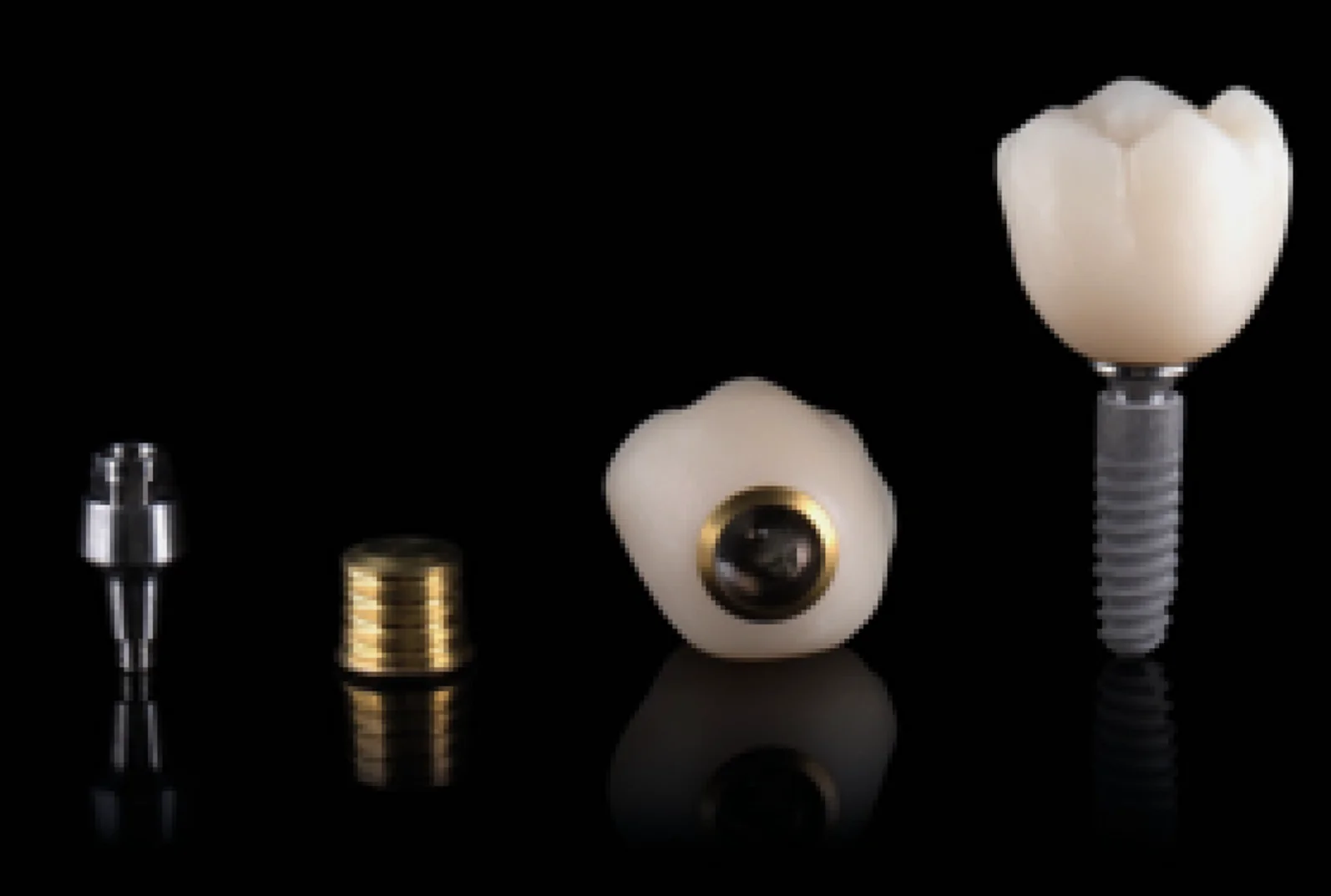
Components of the Acuris System (from left to right): Conometric titanium Acuris abutment, titanium nitride (TiN)-coated coping, extraorally luted full-ceramic crown on TiN coping, and the fully seated crown–abutment–implant assembly

This investigation was designed as a prospective, multicenter, non-interventional study evaluating the clinical performance of a conometric retention concept for single implant restorations using a purpose-designed abutment system (Acuris, Dentsply Sirona Implants). The investigated retention mechanism is based on a conical abutment with a 5.9° taper and a titanium nitride–coated coping serving as the female component of the conometric interface. Figures 1 and 2 illustrate the retention principle and system components.

The study was conducted in nine clinical centers in Europe (Germany, Italy, Spain, and the United Kingdom) and North America (Canada and the United States), including both private practices and university-based institutions. Three commercially available implant systems were employed in the study (Ankylos, AN; Astra Tech Implant System, AT and Xive, XI, all Dentsply Sirona Implants). Each center was assigned one implant system, while the prosthetic concept and workflow were standardized across all sites. Table 1 shows the number of implants per center.

**Table 1:** Implant type per system and center

Center	Implant system	Central incisor	Lateral incisor	Canine	Premolar	Molar	Total n = 144
Site 1	Ankylos	1 (4.5%)	0 (0%)	1 (4.5%)	5 (22.7%)	15 (68.2%)	22
Site 2	Ankylos	0 (0%)	0 (0%)	0 (0%)	10 (58.8%)	7 (41.2%)	17
Site 3	Ankylos	1 (5.3%)	1 (5.3%)	0 (0%)	9 (47.4%)	8 (42.1%)	19
Site 4	Astra Tech	0 (0%)	1 (14.3%)	0 (0%)	1 (14.3%)	5 (71.4%)	7
Site 5	Astra Tech	1 (4.2%)	1 (4.2%)	0 (0%)	7 (29.2%)	15 (62.5%)	24
Site 6	Astra Tech	0 (0%)	0 (0%)	1 (3.8%)	8 (30.8%)	17 (65.4%)	26
Site 7	Xive	0 (0%)	0 (0%)	1 (11.1%)	4 (44.4%)	4 (44.4%)	9
Site 8	Xive	0 (0%)	1 (9.1%)	0 (0%)	10 (90.9%)	0 (0%)	11
Site 9	Xive	0 (0%)	0 (0%)	0 (0%)	9 (100.0%)	0 (0%)	9

Values are reported as n, with the percentage of implants within each center provided in parentheses

Data was collected prospectively using standardized documentation. The study visits included the following: screening, implant placement, post-operative check, impression taking, delivery of the permanent restoration, and follow-up at 6 months and one year after permanent restoration.

Each participating study center was represented by a single clinician who was responsible for all implant treatments within that site. To ensure consistency across centers, all participating clinicians underwent a joint calibration session before study initiation. During this training, examination procedures, surgical and prosthetic protocols, and assessment criteria were standardized, including the use of identical instrumentation and uniform operative techniques. The study complied with STROBE guidelines and was conducted in accordance with the Declaration of Helsinki, ISO 14155:2011, and all applicable regulatory requirements.

### Participants

Subjects requiring replacement of a single tooth in the maxilla or mandible (excluding third molars) were screened for eligibility. Inclusion criteria comprised age between 18 and 75 years, indication for a single implant, and provision of written informed consent. Subjects who accepted the invitation had to sign the written informed consent. The screening process entailed clinical and radiographic examinations in accordance with standard pre-implant protocols. Case selection was based on predefined clinical and anatomical criteria to ensure appropriate use of the conometric retention system. Eligible implant sites were required to be adjacent to teeth with natural roots or implant-supported restorations; however, in cases involving second molar sites, a distal edentulous space was considered acceptable. In addition, subjects were required to present with functional occlusion in the opposing jaw, supported by a natural tooth, removable prosthesis, or implant-supported restoration. A minimum vertical restorative space of 7 mm was required to accommodate the conometric interface and allow reliable friction-based retention, in accordance with the manufacturer’s recommendations. Anatomical limitations included unfavorable implant angulation exceeding the compensatory range of available angulated abutments, restricted interocclusal clearance, and inadequate soft-tissue conditions preventing stable seating of the prosthetic components. Contraindications for the use of the conometric system included insufficient vertical space and the inability to achieve precise seating of the restoration. Exclusion criteria included uncontrolled oral pathology, malignancy, recent radiation therapy in the head and neck region, systemic conditions affecting healing (e.g., uncontrolled diabetes), use of medications impairing osseointegration, need for major bone augmentation, substance abuse, and smoking of more than 10 cigarettes per day.

Ethical approval was obtained individually at each participating site according to local regulations. Thus, approvals were received from the Ethics Committee in Mainz, Germany (file number 2019–14210, June 11, 2019), Sette Laghi, Italy (file number C-OT-17-003, September 10, 2019), Oviedo, Spain (file number C-OT-07-003, May 29, 2019) and (file number C-OT-07-003-27/19, May 20, 2019), London, United Kingdom (file number 19/LO/1831, November 25, 2019), Aurora Ontario, Canada (file number SSU00083815, September 25, 2019) and (SSU00102484, September 27, 2019 for Oklahoma), and Chicago, Illinois, United States, (2019–1044, December 9, 2019). The study was registered at ClinicalTrials.gov, ID, NCT04063878, August 20, 2019.

### Surgical procedures

Implants were placed in healed ridges or fresh extraction sockets according to the manufacturer’s recommendations. One-stage or two-stage surgical protocols were applied based on clinical judgment and primary implant stability. Immediate or conventional loading protocols were used as clinically indicated. Subjects received local anesthesia and postoperative care according to local standards. Antibiotic prophylaxis and chlorhexidine rinses were prescribed at the investigator’s discretion. One to 2 weeks after implant placement, the subject returned for clinical examination of the implant/abutment and the healing process. Any sutures were removed and the subject was given oral hygiene instructions. Adverse events and adverse device effects were recorded continuously throughout the course of the study.

### Prosthetic procedures

**Fig. 3:**
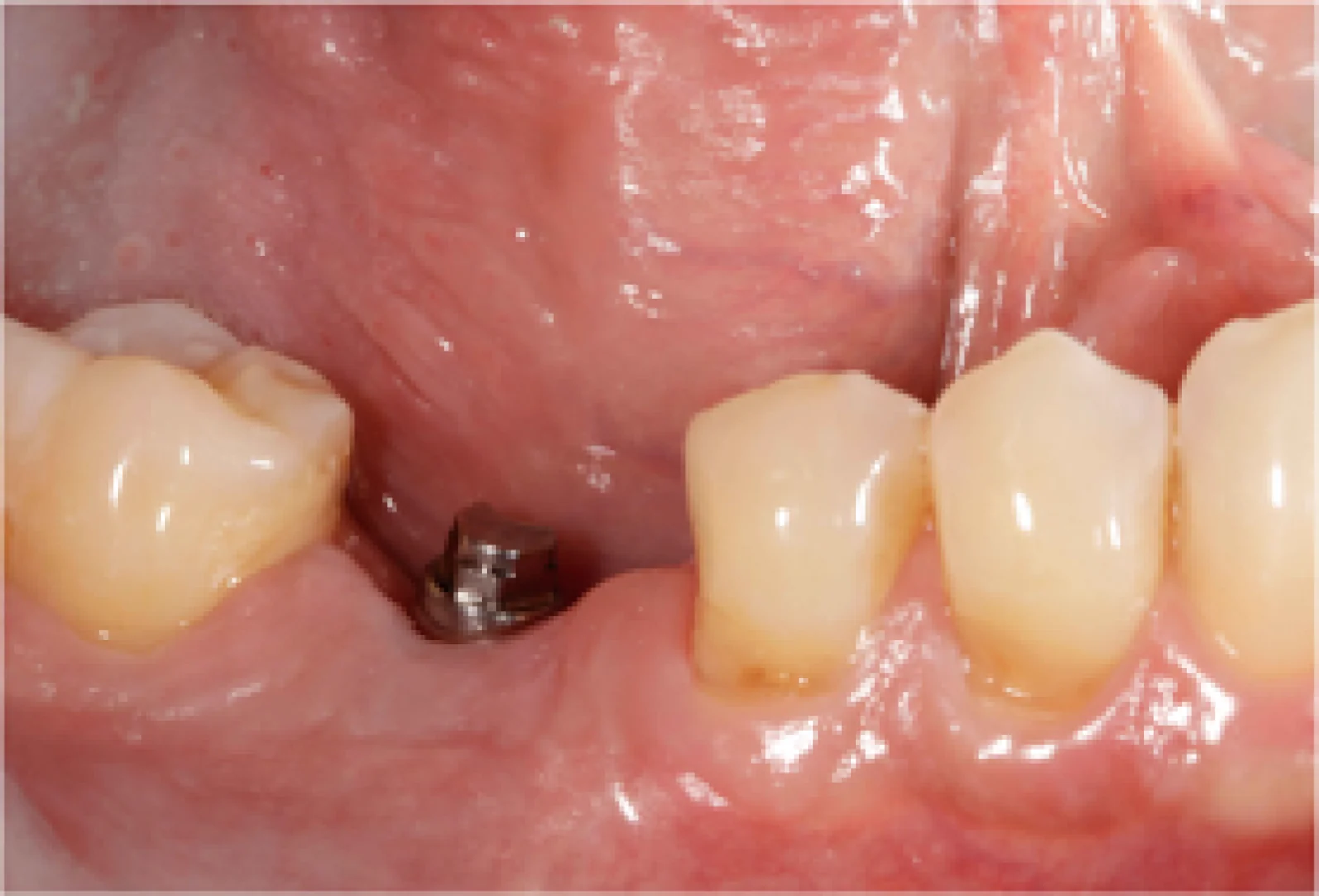
Conometric abutment in place for the replacement of the mandibular right first molar

**Fig. 4:**
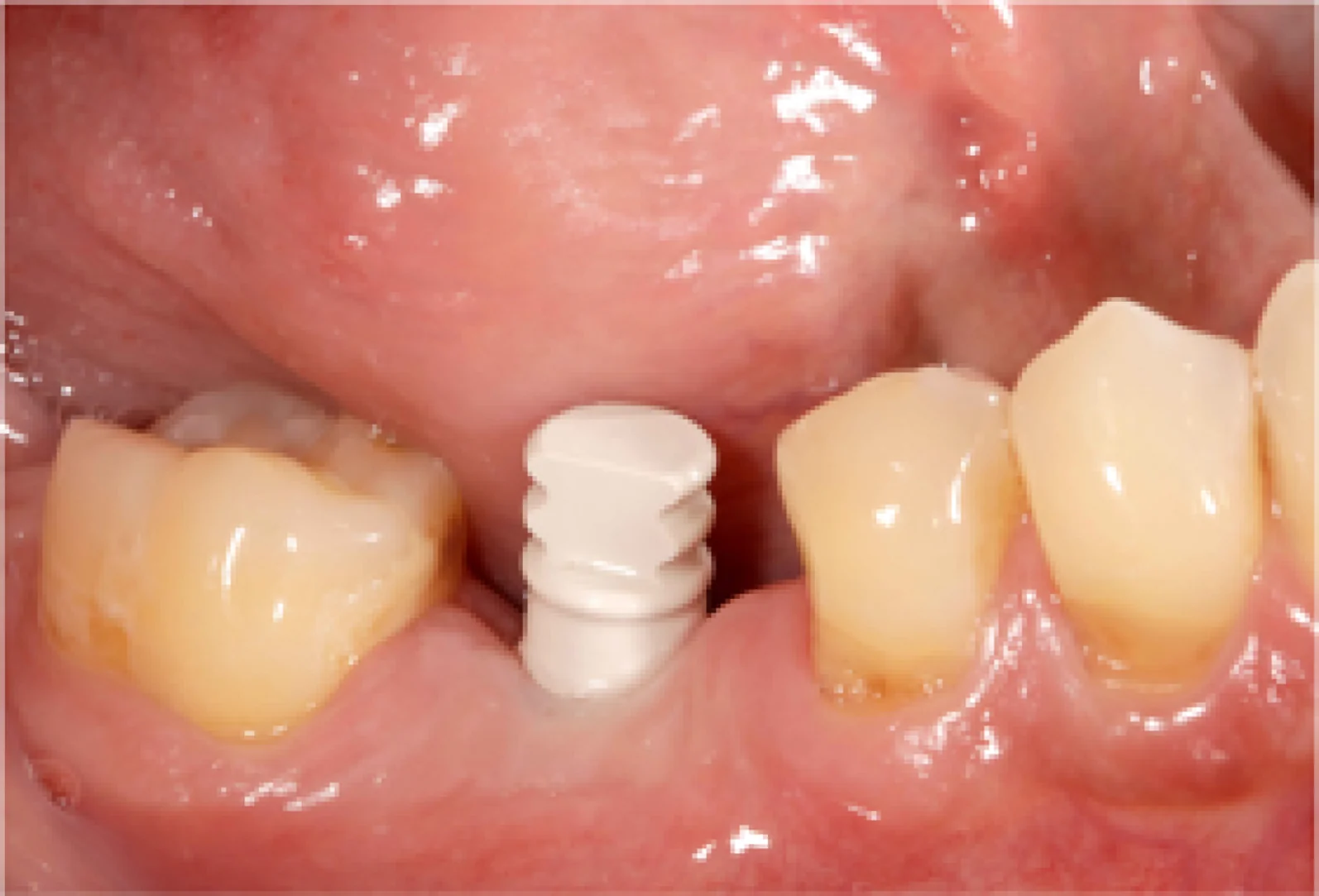
Corresponding PEEK impression coping seated on the conometric abutment prior to analog impression taking

**Fig. 5:**
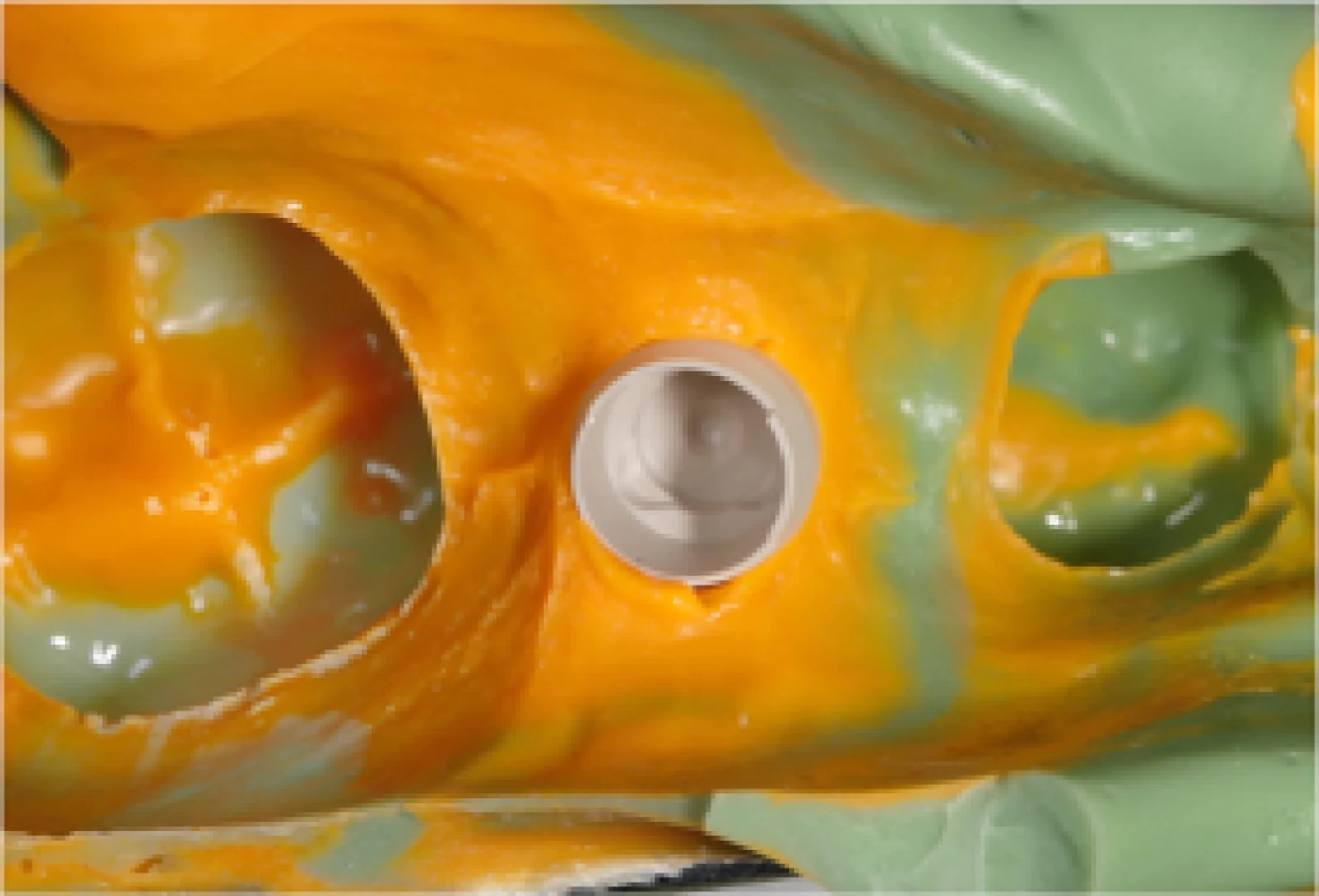
PEEK impression coping picked up in silicone impression using an individual custom tray

**Fig. 6:**
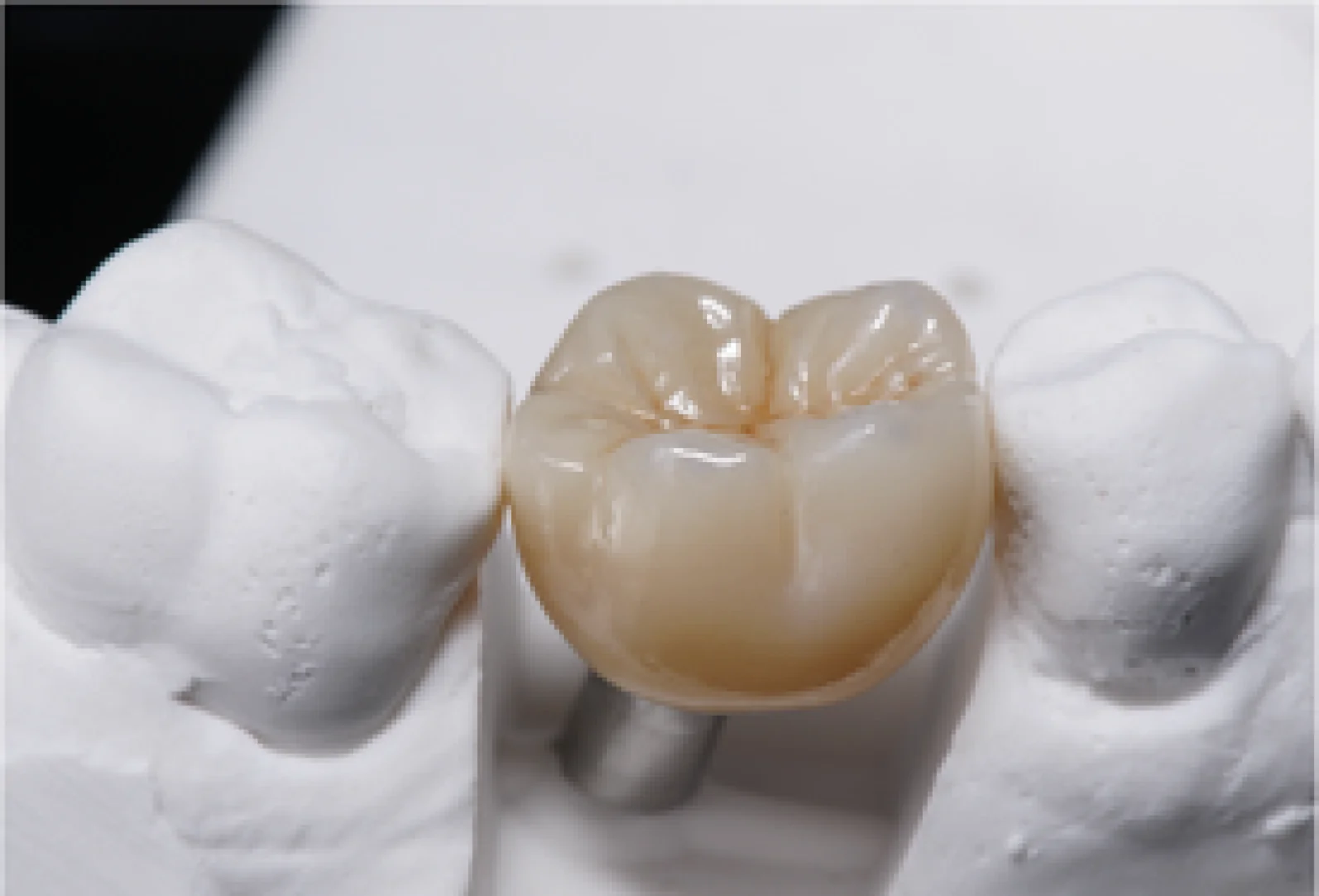
CAD/CAM-fabricated monolithic full-ceramic molar crown on master cast

**Fig. 7:**
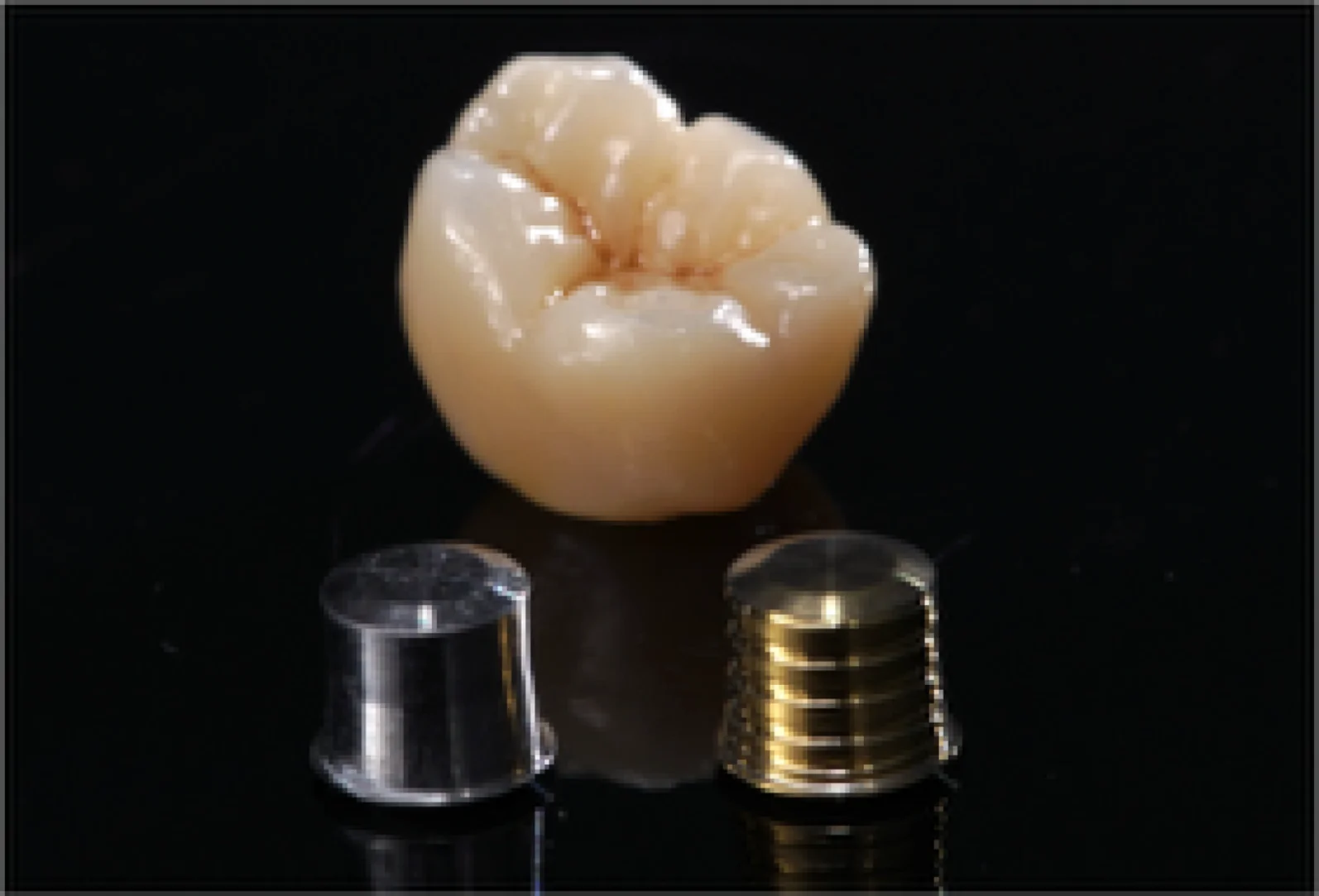
Crown fabricated on a non-precious metal laboratory coping (left) before relining onto the original TiN coping (right)

**Fig. 8:**
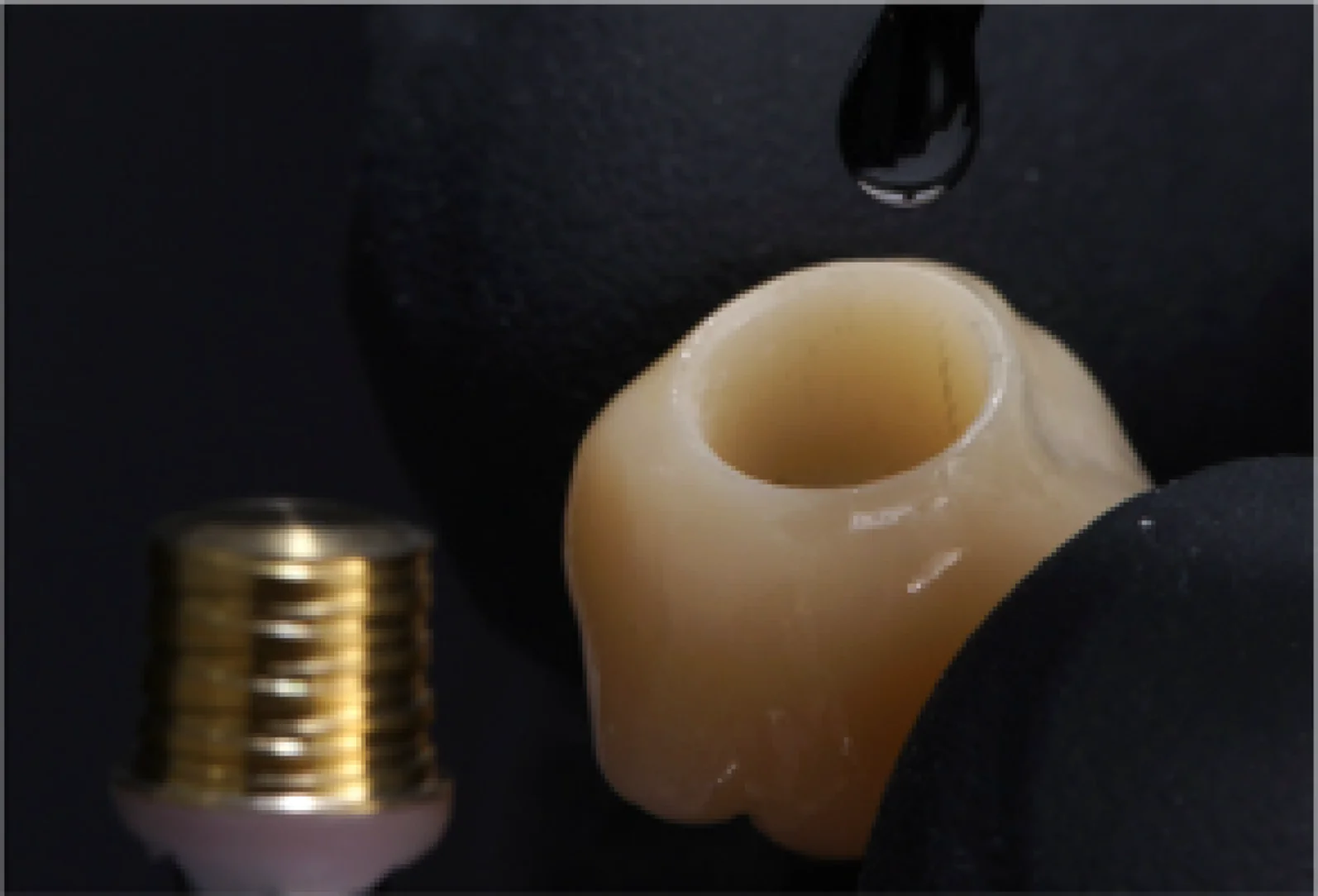
Extraoral luting of all-ceramic crown onto the TiN-coated friction coping using adhesive resin cement

**Fig. 9:**
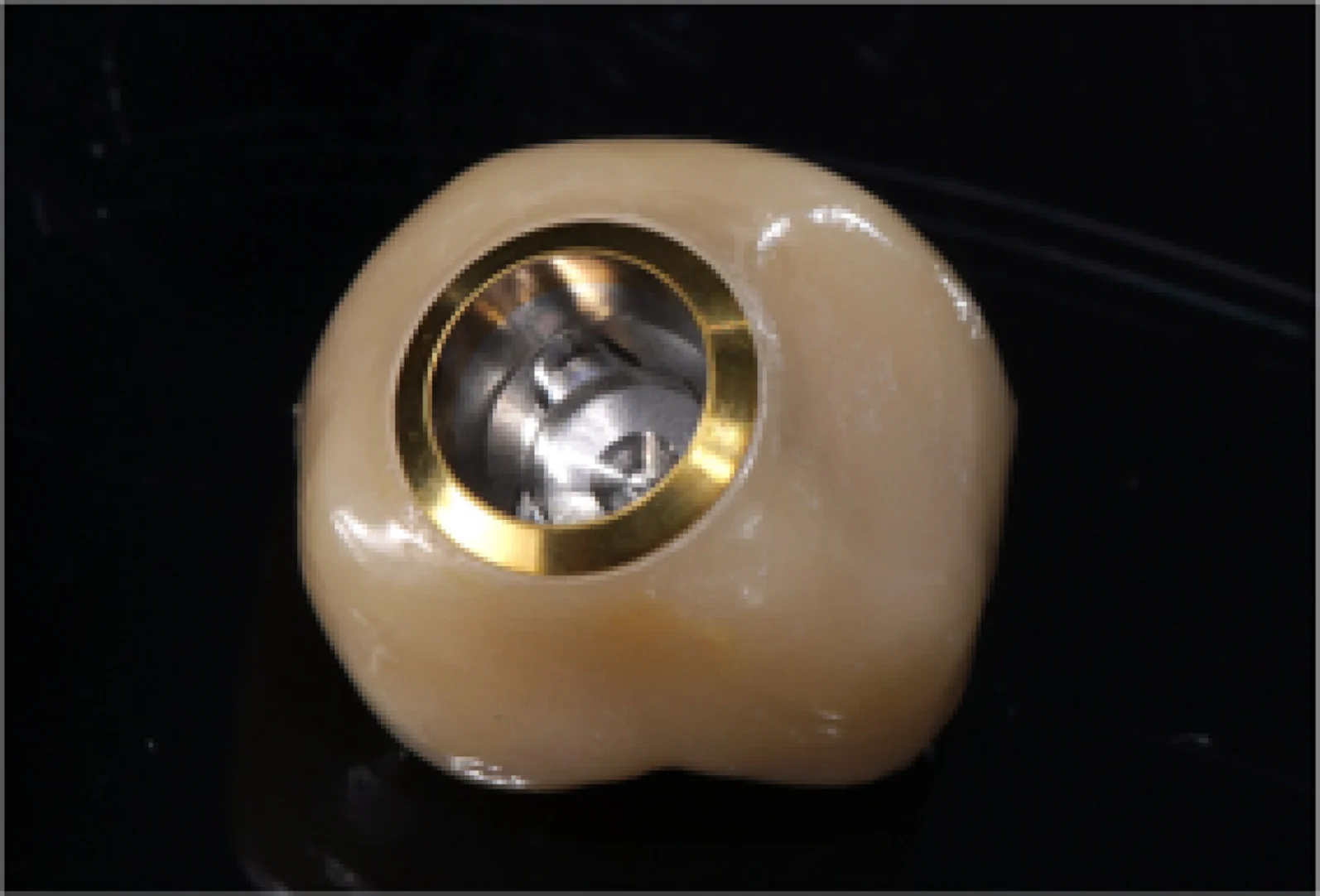
Crown–coping complex after extraoral adhesive bonding

**Fig. 10:**
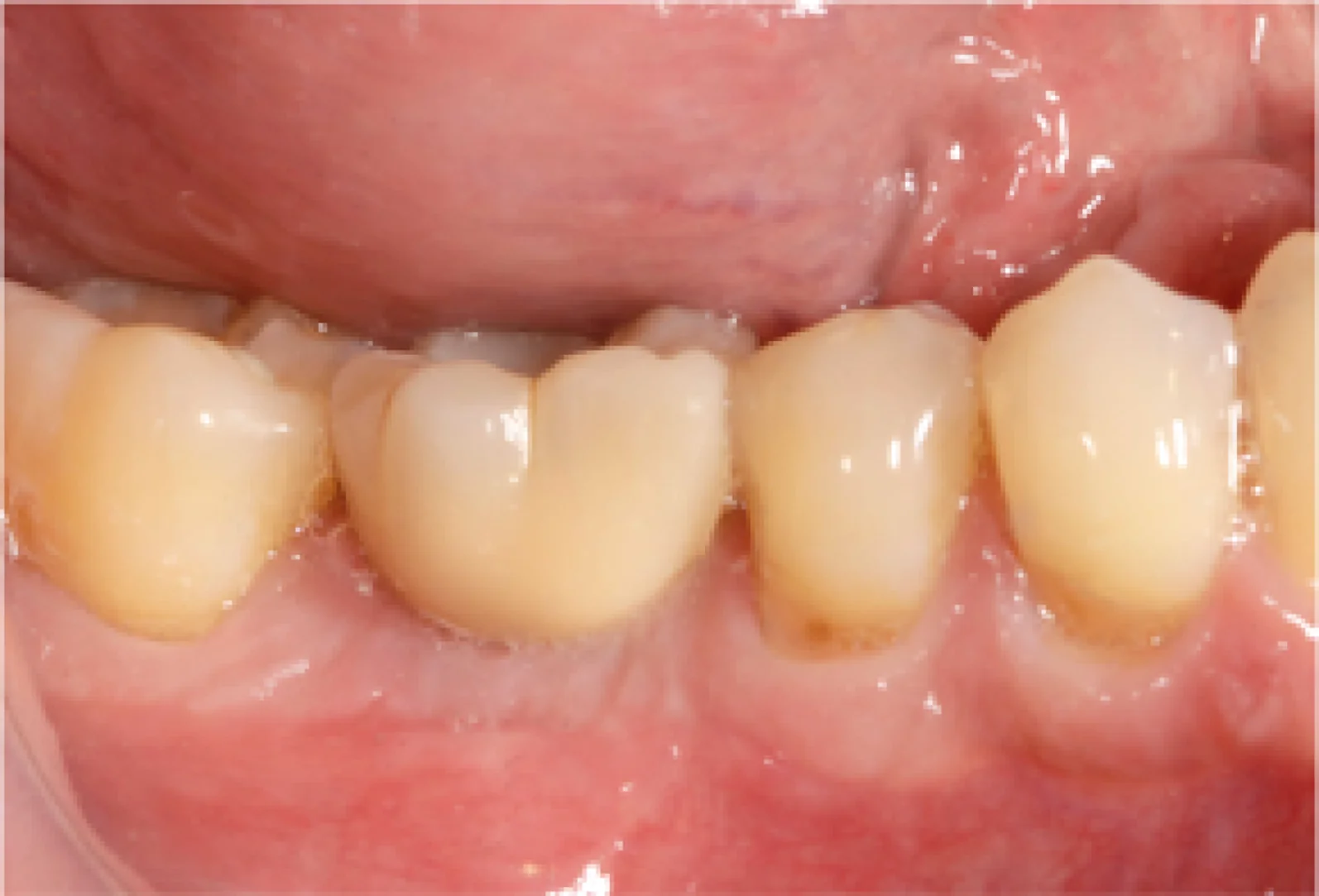
Conometrically seated crown–coping complex in situ after fixation with dedicated tool

**Fig. 11:**
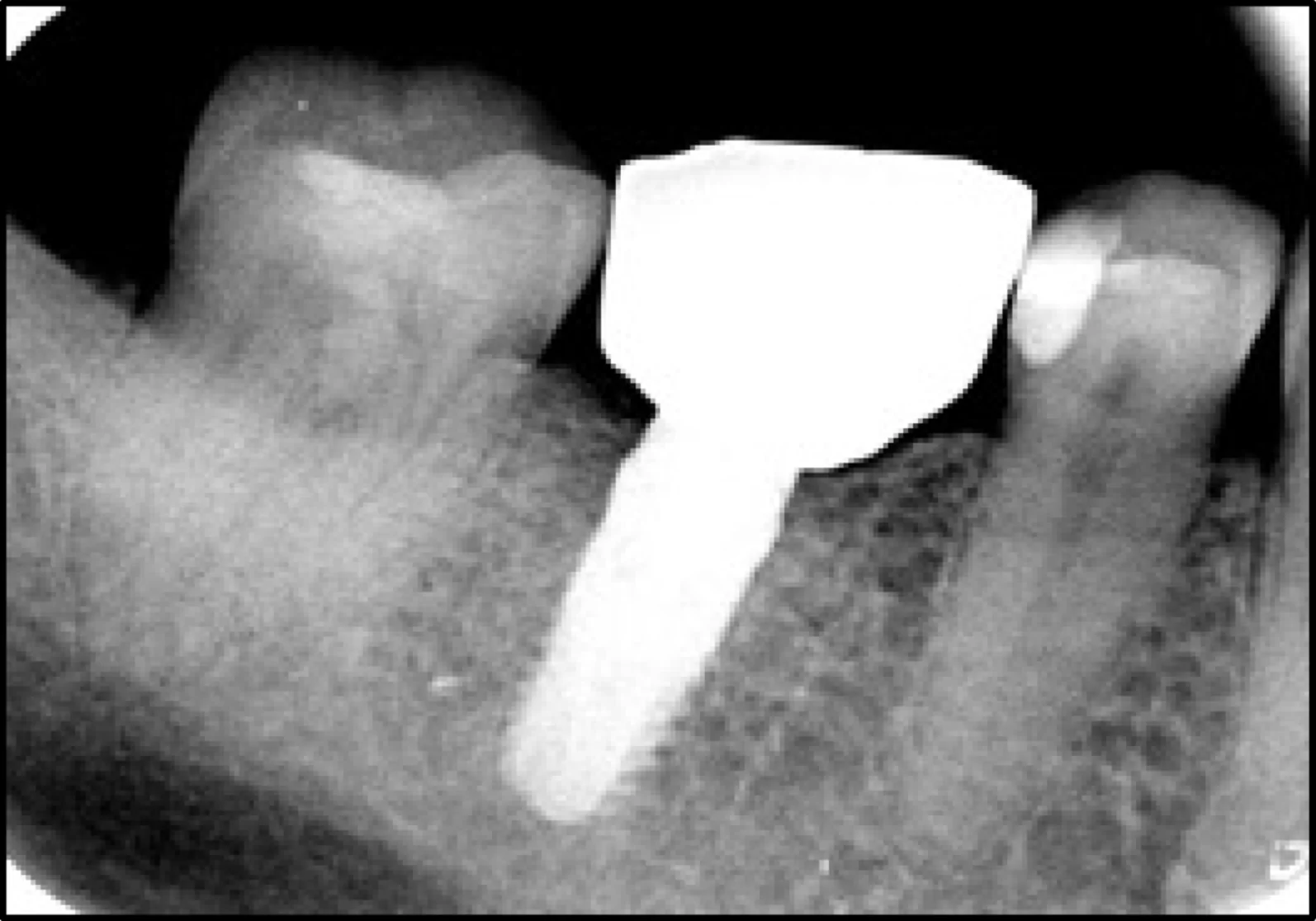
Radiographic confirmation of complete seating of the crown–coping complex on the conometric abutment

Impressions for fabrication of the final ceramic crowns were obtained once implant stability had been confirmed, in accordance with the selected loading protocol. The study was not designed to compare loading strategies, and no stratified analysis was performed. The impressions were taken on conometric abutment level, meaning that in subjects receiving cover screws or abutment/gingiva formers at implant surgery, these were replaced with conometric abutments before impression. For subjects receiving conometric abutments at surgery, the temporary crown or the conometric healing caps, respectively, were removed before impression. All procedures were carried out in accordance with the respective product manuals, catalogues, and the manufacturers’ recommendations. The impression together with the conometric final cap was sent to the laboratory for fabrication of the final crown (Figs. 3, 4, 5, 6, 7, 8, 9, 10, 11).

Prior to delivery, the final all-ceramic crown was extraorally luted to the prefabricated titanium nitride (TiN) -coated coping under controlled laboratory conditions. Although individual restorative materials and adhesive systems varied slightly between participating centers, all sites followed the same adhesive bonding protocol recommended for the respective ceramic and luting materials. In general, the internal bonding surface of the crown was conditioned and treated with a ceramic primer before adhesive luting to the TiN coping using a resin-based cement. Excess luting material was removed after initial polymerization, and the margins were protected from oxygen inhibition according to the respective manufacturer’s recommendations. Final polymerization was completed extraorally before clinical delivery of the crown–coping complex, and the adhesive interface was subsequently finished and polished. At the clinical appointment, the resulting crown–coping complex was conometrically seated onto the abutment using the dedicated fixation tool (Dentsply Sirona Implants). Proper seating of the conometric crown–coping complex was verified clinically prior to radiographic confirmation. Seating was assessed by tactile feedback during insertion, absence of rotational or vertical mobility, and visual inspection of the marginal fit at the peri-implant soft-tissue level. No additional specialized instruments were required beyond the manufacturer-provided dedicated fixation tool. The clinical delivery protocol followed a standardized stepwise sequence: removal of provisional components, cleaning and inspection of the abutment, conometric seating of the crown–coping complex using the fixation tool, clinical verification of complete seating, and subsequent radiographic confirmation. Only restorations demonstrating complete seating were accepted for final delivery. A clinical examination was performed to assess the peri-implant mucosa, including probing pocket depth (PPD), plaque (PI), and bleeding on probing (BoP). Oral hygiene instructions were reinforced if necessary.

### Outcome measures

Primary outcomes were implant survival and prosthetic survival. Implant survival was defined as the implant remaining in situ, whereas prosthetic survival was defined as maintenance of the restoration without irreversible loss of conometric retention. Secondary outcomes included marginal bone level (MBL) changes, peri-implant soft tissue conditions (bleeding on probing, BOP), plaque index (PI), probing pocket depth (PPD), and the occurrence of biological and technical complications.

Intraoral periapical radiographs were obtained at implant placement (IP), at delivery of the permanent restoration (PR), and at the 1-year follow-up after prosthetic loading (PR + 1 year). For radiographs obtained at delivery of the permanent restoration and at the 1-year follow-up, the Acuris abutment and the crown–TiN coping complex were in situ. Radiographs were acquired using a standardized paralleling technique whenever clinically feasible. Only radiographs with clearly visible interproximal implant threads and identifiable reference landmarks were included for analysis.

**Fig. 12:**
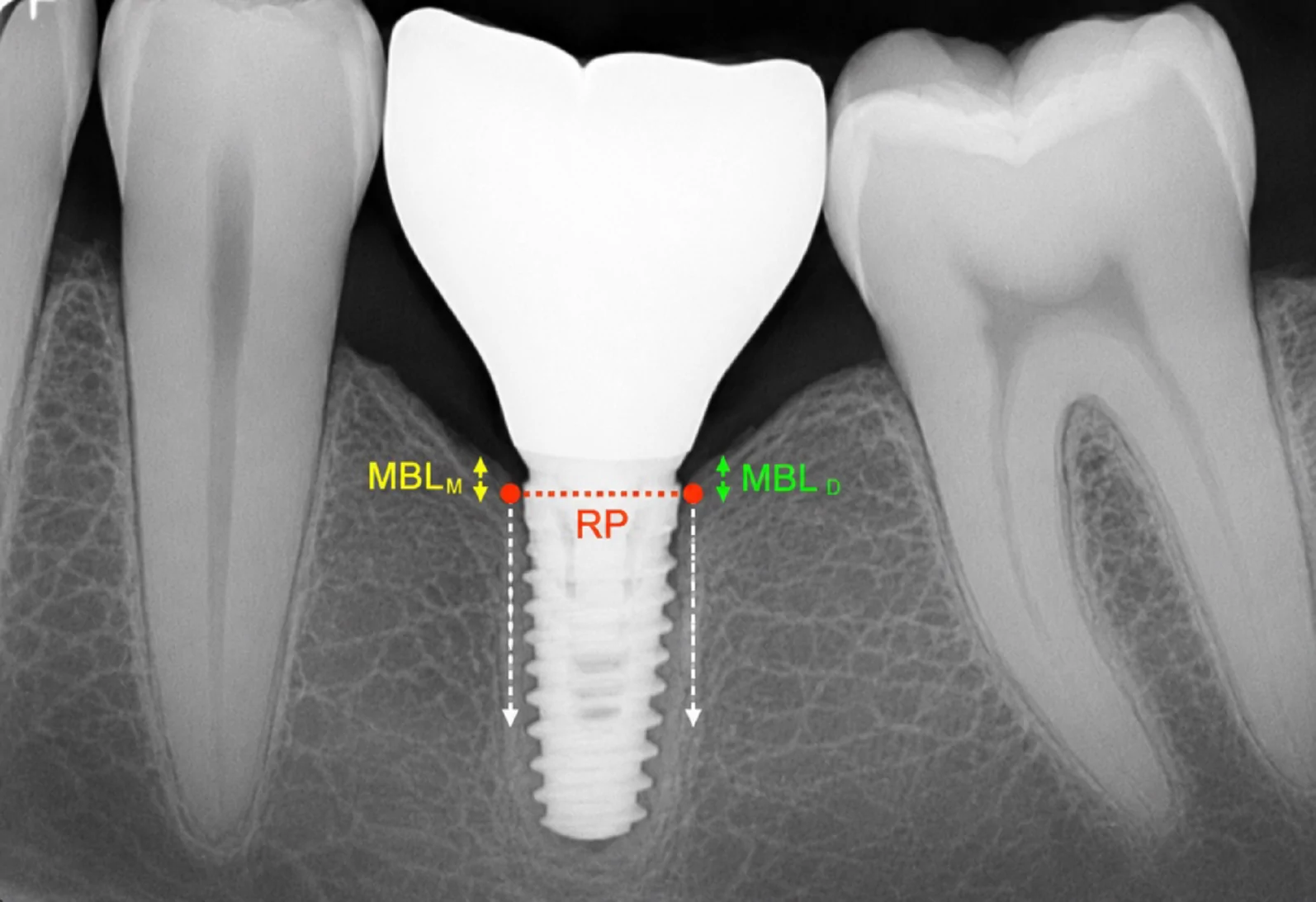
Representative radiograph illustrating the marginal bone level measurement methodology. The reference point (RP) was defined as the junction between the roughened implant surface and the machined bevel at the implant platform. Mesial (MBL_M_) and distal (MBL_D_) marginal bone levels were measured as the vertical distance from the reference point to the most coronal bone-to-implant contact, parallel to the long axis of the implant

All radiographic measurements were performed by a single independent external radiologist who was not involved in the clinical treatment. Marginal bone levels were assessed using calibrated digital imaging software (Illustrator CS, Adobe Systems) on a 24-inch LCD screen (iMac, Apple) with a screen resolution of 1,920 × 1,200 pixels. Calibration was performed using the known implant dimensions. A predefined implant-related reference point, defined as the junction between the roughened implant surface and the machined bevel at the implant platform, was used consistently for each implant system. This landmark was selected because it could be identified consistently across all implant designs, irrespective of implant geometry or insertion depth. Measurements were recorded to the nearest 0.1 mm. The vertical distance from the reference point to the most coronal bone-to-implant contact was measured separately at the mesial and distal aspects, parallel to the long axis of the implant Fig. 12 shows a representative radiograph illustrating the marginal bone level measurement methodology.

Complications were documented prospectively and categorized as reversible or irreversible. Loss of conometric retention was classified as either reversible or irreversible. Reversible loss of retention was defined as events in which retention could be successfully re-established by clinical management without replacement of the implant-supported restoration or abandonment of the conometric retention concept. Reversible retention events were recorded as technical complications and did not affect prosthetic survival. Irreversible loss of retention was defined as failure to restore stable conometric retention despite corrective measures, requiring conversion to an alternative prosthetic solution. Only irreversible loss of retention was considered a prosthetic survival failure. For the present analysis, complication frequency is reported as the total number of events and as an event rate relative to the per-protocol population.

### Statistical analysis

Statistical analyses were performed using SPSS (IBM Corp.) and Excel (Microsoft). Descriptive statistics and confidence intervals were calculated. Sample size estimation was based on an expected prosthetic survival rate of 97% at 1 year, with a one-sided significance level of 2.5% and a power of approximately 90%, resulting in a required sample size of 133 subjects.

Implant and prosthetic survival rates were calculated with exact binomial confidence intervals. Continuous variables were analyzed using the Wilcoxon signed-rank test, and categorical data were evaluated using binomial tests. P-values were reported descriptively, and no adjustment for multiple testing was applied.

## Results

**Fig. 13:**
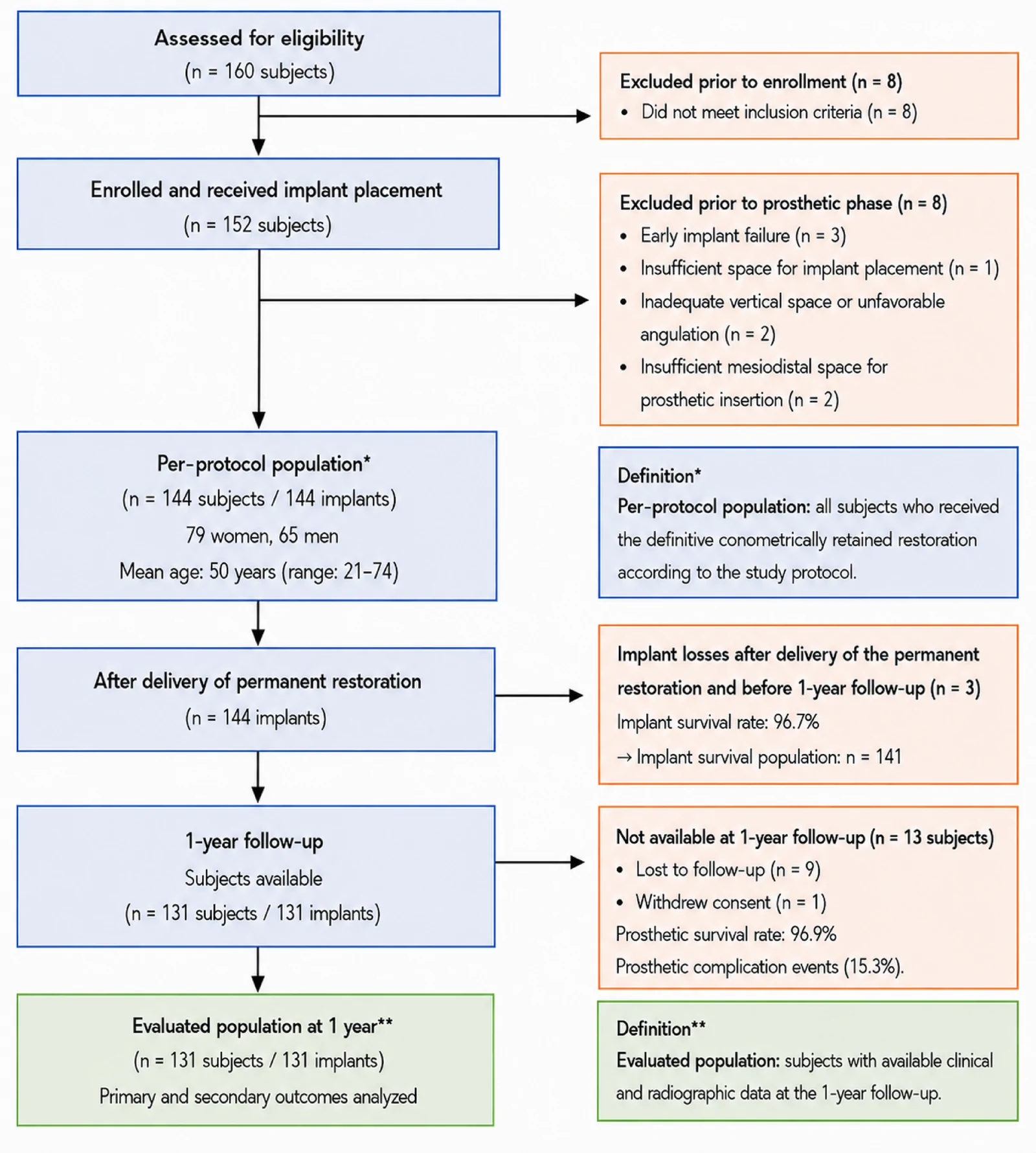
Flowchart of study enrollment, exclusions, analysis populations, and 1-year follow-up outcomes

Between July 2019 and December 2021, 160 subjects were screened for this study. A detailed overview of patient flow and analysis populations is provided in Fig. 13. Eight subjects did not meet the inclusion criteria, and 152 were subsequently enrolled and received implant placement. Of these, eight subjects were excluded prior to the prosthetic phase due to early implant failure (n = 3), insufficient space for implant placement (n = 1), inadequate vertical space or unfavorable angulation preventing abutment placement (n = 2), and insufficient mesiodistal space preventing prosthetic insertion (n = 2). Consequently, a per-protocol population of 144 subjects was available for analysis (Table 1). This group comprised 79 women and 65 men (mean age 50 years; range 21–74 years) (Table 2).

**Table 2:** Baseline population characteristics

	Female	Male	Total
N	79	65	144
Age			
Min	21	30	21
Max	72	74	74
Mean	49.5	51.3	50.3

Implants were distributed across systems as follows: Ankylos (n = 58), Astra Tech (n = 57), and Xive (n = 29). Bone quality and quantity were classified according to established criteria [29, 30]. Details on implant distribution according to bone quality and quantity are provided in Table 3.

**Table 3:** Implant distribution per bone quantity and quality

Bone quality	Bone quantity				
	A	B	C	D	Total
1	9	3	1	0	13
2	19	42	1	0	62
3	20	22	10	1	53
4	7	8	0	1	16
Total	55	75	12	2	144

**Fig. 14:**
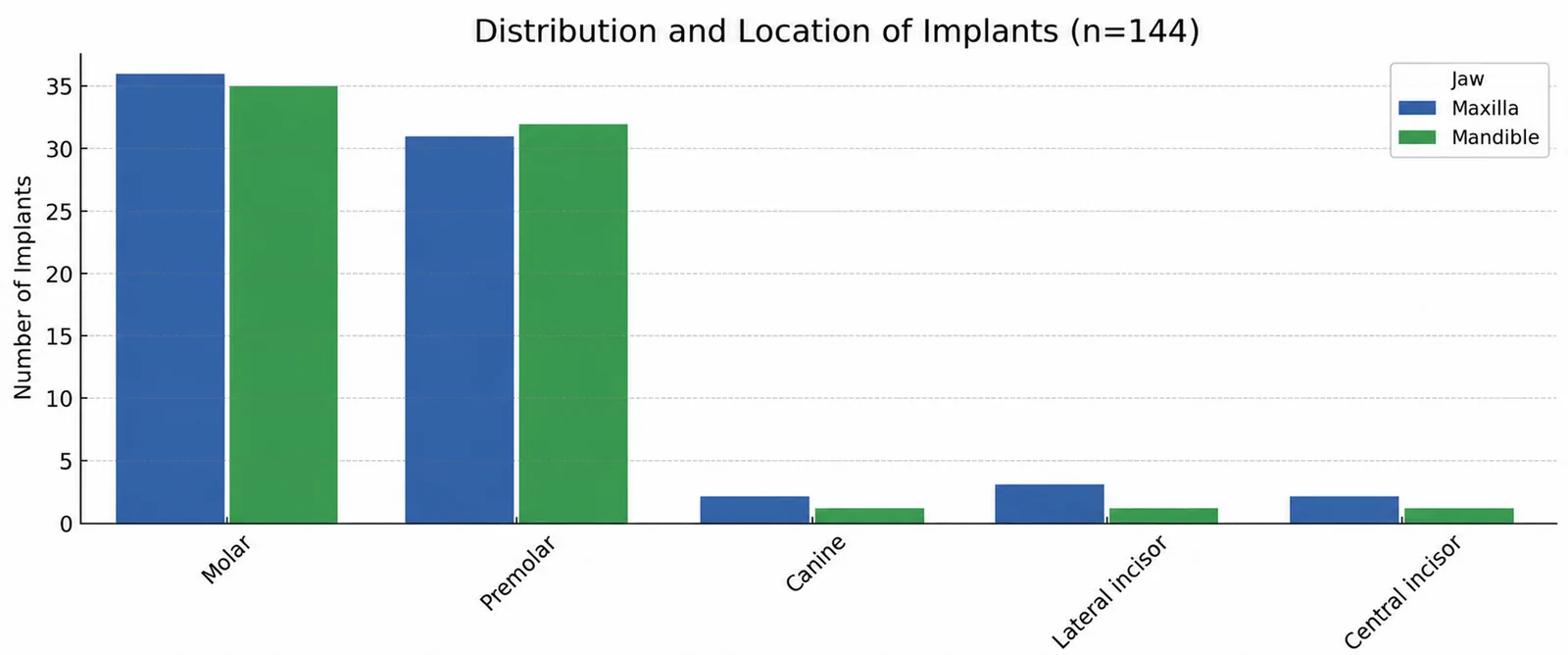
Implant distribution per tooth position (n=144)

Implants were evenly distributed between the maxilla (51.4%) and mandible (48.6%) and were predominantly placed in molar (49.3%) and premolar regions (43.8%). Only 6.9% were placed in anterior positions (Fig. 14).

### Implant and prosthetic survival

Of the 144 implants in the per-protocol population, three implants were lost after delivery of the permanent restoration and before the 1-year follow-up, resulting in an implant survival rate of 96.7%. At 1 year, 131 subjects were available for evaluation (9 lost to follow-up, 1 withdrew consent). Irreversible loss of conometric retention occurred in 4 of 131 restorations between 8 days and 2 months after delivery. In these cases, stable conometric retention could not be re-established despite corrective clinical measures, and conversion to alternative prosthetic solutions was required. These events were therefore classified as prosthetic failures according to the predefined study endpoint. In contrast, reversible retention events were successfully managed clinically without abandoning the conometric retention concept and were recorded as technical complications rather than prosthetic failures. The prosthetic survival rate at 1 year was 96.9% (95% CI: 92.3–99.2%). Because prosthetic survival and implant survival were calculated using different analysis sets, direct comparison of these rates should be interpreted with caution.

### Marginal bone level changes

**Fig. 15:**
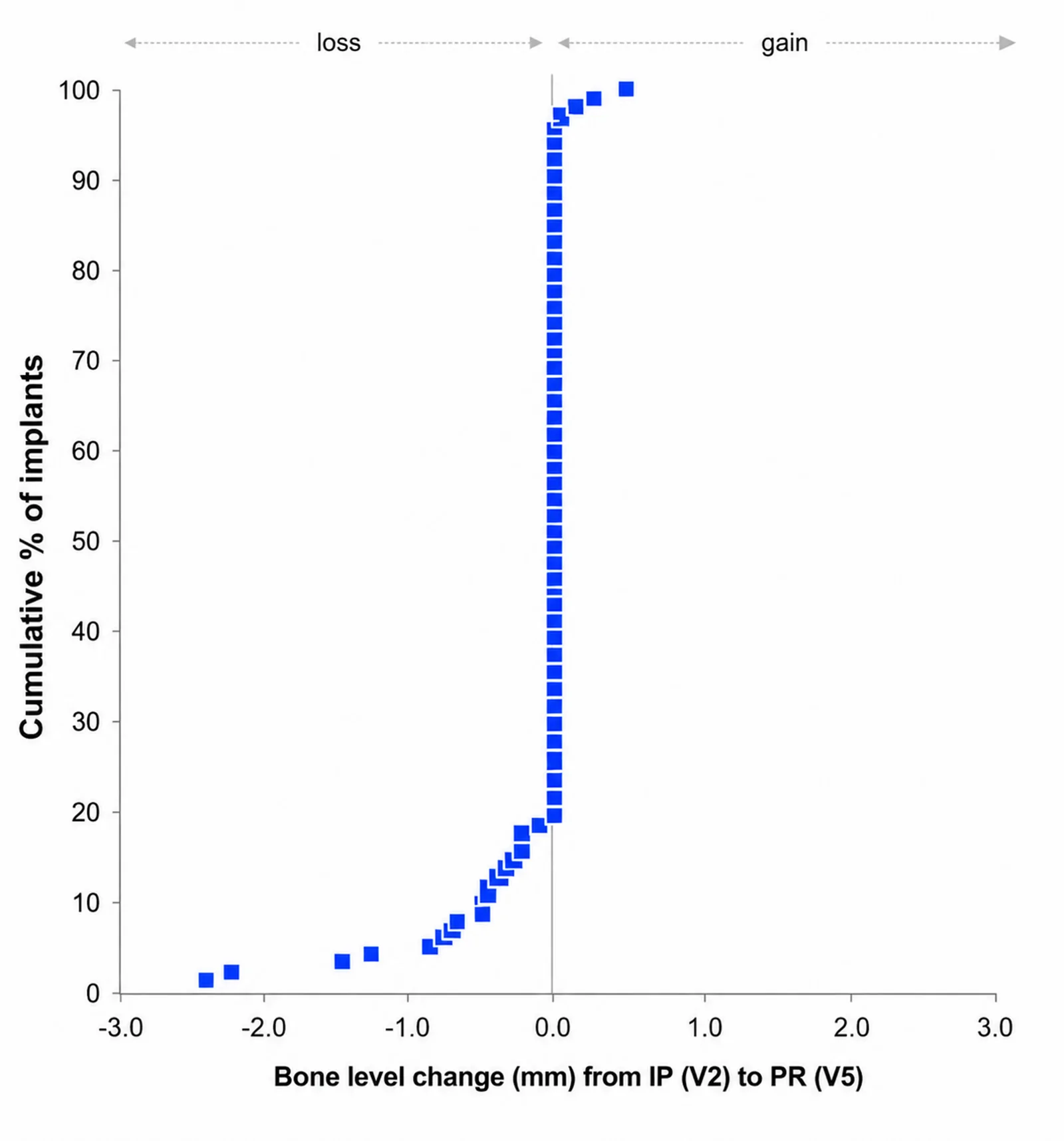
Cumulative distribution of marginal bone level changes: From implant placement (IP) to prosthetic restoration (PR)

**Fig. 16:**
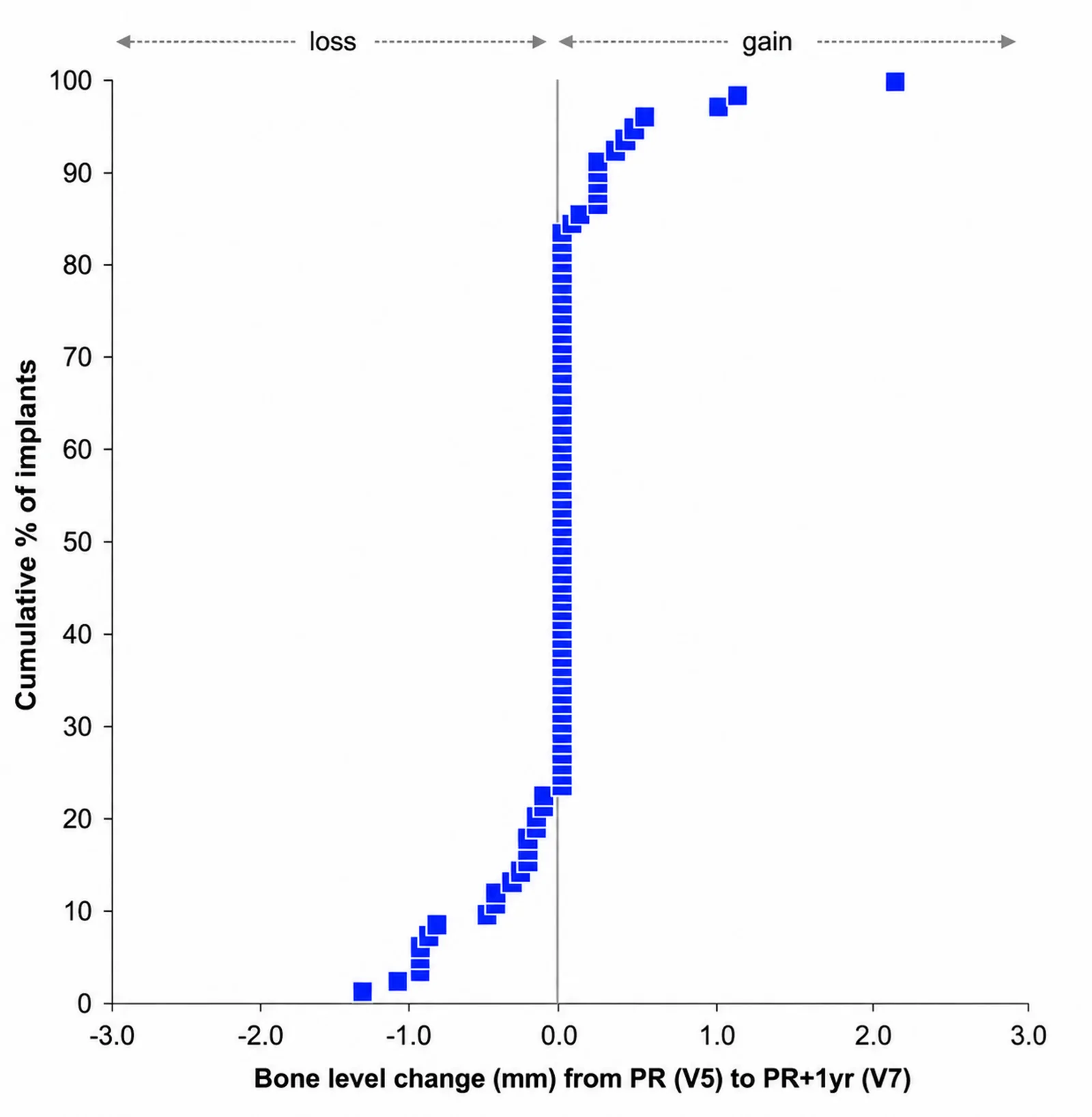
Cumulative distribution of marginal bone level changes: Prosthetic Restoration (baseline) to the 1-year follow-up

Mean marginal bone level (MBL) change from implant placement to permanent restoration was − 0.11 mm (range − 2.35 to 0.70 mm). From permanent restoration to the 1-year follow-up, mean MBL change was − 0.02 mm (range − 1.20 to 2.30 mm) (Table 4; Figs. 15 and 16).

**Table 4:** Marginal bone level changes

	Change from baseline (Gain+/Loss−)		
	PR Baseline IP	PR+1 year Baseline IP	PR+1 year Baseline PR
N	131	119	118
Min	− 2.35	− 1.20	− 1.20
Median	0.00	0.00	0.00
Max	0.70	1.20	2.30
Mean	− 0.11	− 0.11	− 0.02
Sd	0.37	0.31	0.37
95% CI [LL, UL]	[− 0.17, − 0.04]	[− 0.17, − 0.05]	[− 0.08, 0.05]
P-value	0.00016	0.00003	0.42501

*IP* Implant placement, *PR* Permanent restoration

### Peri-implant soft tissue conditions

Bleeding on probing was present in 15.4% of implants at delivery of the permanent restoration, increasing to 31.8% at 6 months and 44.1% at 1 year (Table 5). Plaque levels remained stable between 6 months (24.2%) and 1 year (24.4%). Mean probing pocket depth changed by −0.12 mm (range − 2.00 to 1.25 mm) at 6 months and − 0.32 mm (range − 3.50 to 1.50 mm) at 1 year (Table 6).

**Table 5:** Condition of peri-implant mucosa (N=144)

	PR	PR+6 months	PR+1 year
N subjects	143	132	127
Missing	1	12	17
*Bleeding (BOP)*			
No	121	90	71
Yes	22	42	56
(%)			
No	84.6%	68.2%	55.9%
Yes	15.4%	31.8%	44.1%
P-value	N/A	0.00191	0.00000
N subjects		132	127
*Plaque (PI)*			
No		100	96
Yes		32	21
(%)			
No		75.8%	75.6%
Yes		24.2%	24.4%
P-value		N/A	0.82380

*PR* Permanent Restoration

**Table 6:** Probing pocket depth

	Probing Pocket Depth (PPD)—Implant mean				
	PR	PR+6 months	PR+1 year	Change from baseline (PR) (Pocket decrease+/Increase−)	
				PR+6 months	PR+1 year
N	143	132	127	131	125
Min	1.00	1.00	1.00	− 2.00	− 3.50
Max	5.25	5.25	5.00	1.25	1.50
Mean	2.10	2.22	2.43	− 0.12	− 0.32
Sd	0.85	0.91	0.91	0.66	0.86
95% CI [LL, UL]	[1.96, 2.24]	[2.06, 2.38]	[2.27, 2.59]	[− 0.24, − 0.01]	[− 0.47, − 0.17]
P-value	N/A	N/A	N/A	0.05563	0.00007

*PR* Permanent Restoration

### Prosthetic complications

During the first year, 22 prosthetic complication events were recorded (15.3%). Of these, 18 were reversible technical complications (12.5%), including abutment loosening/dislodgement (n = 9) and loss of crown retention (n = 9), all managed without replacement of the restoration (Table 7). In addition, 4 irreversible losses of conometric retention occurred (2.8%), requiring conversion to alternative prosthetic solutions. Irreversible retention loss occurred in Ankylos (n = 2) and Xive (n = 2) implants, while no such events were observed in Astra Tech implants. Given the limited number of events, no system-specific pattern could be identified (Table 8).

**Table 7:** Prosthetic complications during the first year after delivery of permanent restoration

Complication type	n	% (per-protocol population, n = 144)	Management
Abutment loosening/dislodgement (reversible)	9	6.3	Clinical correction/re-seating
Loss of crown retention (reversible)	9	6.3	Clinical re-seating/correction
Irreversible loss of conometric retention	4	2.8	Conversion to alternative prosthetic solution
Total prosthetic complication events	22	15.3

**Table 8:** Distribution of irreversible retention loss by implant system

Implant system	n implants	n failures	%
Ankylos (AN)	58	2	3.4
Astra Tech (AT)	57	0	0.0
Xive (XI)	29	2	6.9

## Discussion

The present prospective multicenter study evaluated the clinical performance of a conometric retention system for single implant restorations after one year of function. The results demonstrated high implant survival, acceptable short-term prosthetic performance, and minimal marginal bone level changes despite a clinically relevant incidence of predominantly manageable technical complications. Implant survival was 96.7% and prosthetic survival 96.9% at 1 year. However, irreversible loss of conometric retention occurred in 3.05% of cases and required conversion to alternative prosthetic solutions. Although implant and prosthetic survival rates were high, the observed prosthetic complication rate of 15.3% after 1 year was higher than rates commonly reported for conventional screw- and cement-retained single implant restorations [31–34]. In particular, retention-related complications occurred more frequently and should therefore be considered a clinically relevant limitation of the investigated conometric retention concept.

Therefore, the initial hypothesis of complication rates comparable to conventional retention concepts could not be confirmed.

Previous studies have reported favorable short-term outcomes for conometric systems, including stable peri-implant tissues and reliable retention in clinical settings, as well as mechanical stability demonstrated in experimental studies [25–27, 35]. A recent systematic review and meta-analysis by De Angelis et al. demonstrated high implant and prosthetic survival rates across different conometric applications [36]. However, the included studies were heterogeneous and provided limited data on single implant restorations. The present multicenter data therefore contribute clinically relevant evidence for this specific indication.

Marginal bone remodeling observed in the present study was minimal and compares favorably with reported outcomes for conventional retention concepts [37, 38]. The absence of residual cement and reduced microbial colonization at the conometric interface may contribute to this favorable bone response, as suggested by previous in vitro studies [39, 40].

Peri-implant soft tissue conditions showed a progressive increase in bleeding on probing over time, whereas plaque levels remained stable. The increase in bleeding on probing observed during the first year may indicate early inflammatory changes at the peri-implant soft tissues and should therefore be interpreted with caution despite the limited marginal bone remodeling. At the same time, this finding may reflect multifactorial influences, including soft tissue adaptation, probing variability, and patient-related factors, rather than a direct effect of the retention concept alone. Similar observations have also been reported for other implant-supported restorations [41]. To the authors’ knowledge, this is the first prospective multicenter study to report on the Acuris conometric concept for single implant restorations, underscoring the clinical relevance of the findings.

No clear pattern of complications was observed among the different implant systems used. Although irreversible retention loss occurred only in Ankylos and Xive implants, the small number of events does not allow conclusions regarding system-specific effects.

The overall prosthetic complication rate observed in the present study was higher than that reported in the previously published single-center study by Degidi et al. [28]. Several factors may account for this difference. In contrast to the highly standardized conditions of a single-center investigation, the present study was conducted across multiple international centers, including both university-based institutions and private practices, and involved different implant systems, loading protocols, and clinical operators. Consequently, the study reflects a broader spectrum of clinical conditions and operator experience, which may have contributed to the higher frequency of technical complications observed.

The prosthetic concept, involving extraoral bonding of the crown to a titanium nitride–coated coping, offers potential advantages, including elimination of excess cement and improved esthetics due to the absence of screw access channels. In addition, the restorations were retrievable using conventional instruments, facilitating clinical maintenance when required.

Nevertheless, several limitations must be acknowledged. Although the multicenter design enhances external validity, it also introduces variability related to operative approaches, clinician-related factors, and documentation practices, which may have influenced outcomes despite standardized guidelines. Furthermore, each participating center used only one implant system according to local clinical routines. Consequently, center effects and implant system effects were completely confounded and could not be analyzed independently. As the study was neither designed nor powered to compare implant systems, any apparent differences between systems should be interpreted with caution and considered exploratory only.

The inclusion of both immediate and delayed loading protocols represents an additional source of clinical heterogeneity and may have influenced treatment outcomes. Likewise, variability in anatomical conditions and prosthetic indications may have affected the feasibility and predictability of conometric restorations in individual cases. Given these factors, no meaningful subgroup analyses could be performed, and no multivariable adjustment for potential confounders was undertaken. Therefore, the present findings should be interpreted primarily as descriptive and exploratory.

Although marginal bone levels were evaluated by an independent radiologist, other clinical parameters, including probing depth and bleeding on probing, were assessed at the individual study sites and may be subject to inter-examiner variability. In addition, minor differences in radiographic acquisition and projection geometry protocols across centers represent an inherent limitation of the multicenter design and should be considered when interpreting marginal bone level changes.

Interpretation of early technical complications should also be undertaken with caution. Early failures may be related to case selection, initial seating, or anatomical factors affecting prosthetic fit. Previous studies suggest that sufficient vertical space, optimal contact geometry, and favorable soft-tissue conditions are critical for successful conometric retention [27, 28]. These observations raise the question of whether conometric systems may require stricter indication criteria than conventional retention methods. The present results suggest that careful case selection, particularly regarding available vertical space and seating conditions, is an important factor for the successful clinical application of conometric retention concepts.

The study was non-randomized, and implant allocation was based on logistical factors rather than true random assignment, which may have introduced center-specific bias. In addition, the absence of a control group limits direct comparison with conventional retention concepts. While the overall sample size was adequate, the limited number of anterior restorations restricts generalizability to esthetically demanding sites. Clinical performance in the anterior region may differ because esthetic, spatial, and prosthetic requirements can be more demanding than in posterior restorations. Furthermore, the present 1-year analysis reflects only the first year of a planned five-year observation period and therefore does not allow definitive conclusions regarding long-term marginal bone stability, peri-implant tissue health, or prosthetic performance. Continued follow-up will be essential to determine whether the favorable short-term outcomes observed in this cohort can be maintained over time. Future controlled studies with extended observation periods are required to evaluate the comparative effectiveness and long-term performance of conometric and conventional prosthetic retention concepts.

## Conclusions

Within the limitations of this 1-year analysis, the investigated conometric retention system demonstrated high implant and prosthetic survival rates as well as minimal marginal bone changes. However, a clinically relevant incidence of technical complications, including irreversible loss of conometric retention, was observed during the first year of function. These findings suggest that conometric retention may represent a clinically applicable alternative retention concept for single implant restorations; however, the observed complication rate, retention-related events, and increasing peri-implant inflammatory parameters require careful clinical consideration and long-term evaluation.

## Data Availability

No datasets were generated or analysed during the current study.

## Abbreviations

AN: Ankylos implant system
AT: Astra implant system
BOP: Bleeding on probing
CI: Confidence interval
IP: Implant placement
IMP: Impression
ISQ: Implant stability quotient
MBL: Marginal bone level
PEEK: Polyetheretherketon
PP: Per-protocol
PR: Permanent restoration
SD: Standard deviation
TiN: Titanium nitride
XI: Xive implant system
